# 586. Let's Have a Chat: How Well Does an Artificial Intelligence Chatbot Answer Clinical Infectious Diseases Questions?

**DOI:** 10.1093/ofid/ofae631.181

**Published:** 2025-01-29

**Authors:** Kathleen Hanrahan, Jeffrey Steele, Robert Seabury, Katie A Parsels, Conan MacDougall, Rachel S Britt, Elias Chahine, Elizabeth W Covington, Jason C Gallagher, Wesley D Kufel

**Affiliations:** SUNY Upstate Medical University, Syracuse, New York; State University of New York Upstate University Hospital, Syracuse, NY; SUNY Upstate University Hospital, Syracuse, NY; State University of New York Upstate Medical University, Syracuse, New York; University of California San Francisco, San Francisco, CA; The University of Texas Medical Branch, Galveston, Texas; Palm Beach Atlantic University Gregory School of Pharmacy, West Palm Beach, FL; Auburn University Harrison College of Pharmacy, AUBURN, Alabama; Temple University, Philadelphia, PA; Binghamton University School of Pharmacy Sciences, Binghamton, NY

## Abstract

**Background:**

ChatGPT is an artificial intelligence tool used by practitioners to answer clinical questions. It’s unknown whether ChatGPT provides quality responses to infectious diseases (ID)-specific questions. This study surveyed ID pharmacist subject matter experts (SMEs) to assess the quality of ChatGPT responses.

**Methods:**

The primary outcome was percentage of ChatGPT responses considered useful. Secondary outcomes were SME’s ratings of correctness, completeness, and safety (C/C/S). One hundred clinically encountered questions by ID pharmacists were assembled and internally validated. Questions were entered into ChatGPT version 3.5 and responses were recorded. Definitions for useful and C/C/S were based on prior definitions and literature. A 0-10 rating scale for C/C/S was developed and validated for interrater reliability using a random sample. Questions with ChatGPT responses were sent to five SMEs for evaluation. Ordinal and categorical variables were assessed for interrater reliability using an average measures intraclass correlation coefficient (ICC) and Fleiss Multirater Kappa (FMK), respectively. SMEs’ responses were compared using the Kruskal-Wallis and Chi-square tests for ordinal and categorical variables, respectively. A post-hoc analysis was performed to identify the location of differences between SME ratings based on question difficulty and category for C/C/S using a Mann-Whitney U test with Bonferroni correction.

**Results:**

SMEs considered 41.8% of responses useful. Median (IQR) ratings for C/C/S were 7 (IQR 4-9), 5 (IQR 3-8), and 8 (IQR 4-10), respectively. The FMK for useful was 0.379 (95% CI 0.317-0.441) indicating fair agreement, and ICC were 0.820 (95% CI 0.758-0.870), 0.745 (95% CI 0.656-0.816), and 0.833 (95% CI 0.775-0.880) for C/C/S, respectively, indicating substantial agreement. No significant difference was observed between SME responses for the percentage of responses considered useful. Neither question category nor difficulty resulted in a difference in SMEs’ ratings for C/C/S or percentage of responses considered useful.

**Conclusion:**

Fewer than half of ChatGPT responses were considered useful by SMEs. However, responses were mostly considered correct and safe, but often deemed incomplete.

**Disclosures:**

**Conan MacDougall, PharmD, MAS**, Merck: Grant/Research Support **Elias Chahine, Pharm.D.**, Seqirus: Advisor/Consultant|Seqirus: Honoraria **Wesley D. Kufel, Pharm.D., BCPS, BCIDP**, Merck & Co.: Grant/Research Support|Shionogi, Inc: Grant/Research Support

